# Chances and Limitations of Wild Bird Monitoring for the Avian Influenza Virus H5N1 — Detection of Pathogens Highly Mobile in Time and Space

**DOI:** 10.1371/journal.pone.0006639

**Published:** 2009-08-14

**Authors:** Hendrik Wilking, Mario Ziller, Christoph Staubach, Anja Globig, Timm C. Harder, Franz J. Conraths

**Affiliations:** 1 Institute of Epidemiology, Friedrich-Loeffler-Institut, Federal Research Institute for Animal Health, Wusterhausen, Germany; 2 Institute of Diagnostic Virology, Friedrich-Loeffler-Institut, Federal Research Institute for Animal Health, Greifswald - Insel Riems, Germany; 3 Institute of Diagnostic Virology, Friedrich-Loeffler-Institut, Federal Research Institute for Animal Health, Greifswald - Insel Riems, Germany; University of Pretoria, South Africa

## Abstract

Highly pathogenic influenza virus (HPAIV) H5N1 proved to be remarkably mobile in migratory bird populations where it has led to extensive outbreaks for which the true number of affected birds usually cannot be determined. For the evaluation of avian influenza monitoring and HPAIV early warning systems, we propose a time-series analysis that includes the estimation of confidence intervals for (i) the prevalence in outbreak situations or (ii) in the apparent absence of disease in time intervals for specified regional units. For the German outbreak regions in 2006 and 2007, the upper 95% confidence limit allowed the detection of prevalences below 1% only for certain time intervals. Although more than 25,000 birds were sampled in Germany per year, the upper 95% confidence limit did not fall below 5% in the outbreak regions for most of the time. The proposed analysis can be used to monitor water bodies and high risk areas, also as part of an early-warning system. Chances for an improved targeting of the monitoring system as part of a risk-based approach are discussed with the perspective of reducing sample sizes.

## Introduction

Since 1997, highly pathogenic avian influenza virus (HPAIV) subtype H5N1 of Asian origin has caused outbreaks among poultry and wild birds in a number of countries in Asia, Europe, and Africa. In Southeast Asia and Egypt the virus became endemic in poultry [1], [2] adversely affecting poultry production in small husbandries and intensive livestock holdings in these regions. In 2005 HPAIV H5N1 also emerged in Europe. In general, cases in wild birds preceded those in poultry holdings in a number of European countries. It has therefore been proposed that infected wild birds introduced the virus in late 2005 or early 2006 [3]. This also sparked fears of a continuous threat through forward-backward transmission of the virus between wild birds and domestic poultry. In addition, there is evidence for an increased risk of the establishment of independent transmission cycles among poultry through subclinically infected domestic ducks [4]. Only recently has a detailed knowledge about the course of infection in certain wild water bird species been obtained [5], [6].

The contribution of poultry and wild birds to the distribution of HPAIV H5N1 is still controversial. Poultry movement and wild bird migration are difficult to assess in a quantitative manner [3]. Many investigators rely on the assumption that the spread of this highly mobile virus is reflected by the spatial distribution of cases. But there are limited analyses on the possibilities and constraints that underlie the monitoring approaches, which may lead to a biased and a distorted image of the disease spread. Insufficient sample sizes and selection bias cause problems in assessing risk factors and in performing parameter estimates regarding the spread of HPAIV H5N1 via poultry and wild birds [7], [8].

**Figure 1:**
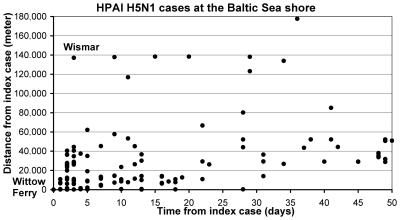
Spatio-temporal pattern of HPAIV H5N1 cases at the Baltic Sea.

The infection dynamics of HPAIV H5N1 in wild birds in Germany in 2006 and 2007 exhibited a specific pattern [9], [10]. All outbreaks were initially connected with water bodies, either seashore or freshwater lakes of various sizes. The epidemic in 2006 among several species of the orders of Anseriformes and Charadriiformes with the epicentre at the coast of the Baltic Sea was the most extended outbreak of HPAIV H5N1 in space and time for wild birds in Europe [9]. Three days after the detection of the first case at the Wittow Ferry on the Isle of Ruegen, a wild duck was confirmed positive for HPAIV H5N1 in the Wismar Bay, 137 km away from the first case on the Isle of Ruegen (Figure 1). Within a few days, infected birds were sampled in various places along the coastline, indicating that the virus was present in the entire region more or less at the same time. After some weeks, the incidence among wild birds decreased at the coastline. A time-space pattern similar to the one observed at the coastline of the Baltic Sea was found at Lake Constance in 2006 [11] and at the Helme reservoir, Berga-Kelbra, where HPAIV H5N1 suddenly emerged in summer 2007 and caused large numbers of lethal infections in wild birds, mainly in Black-necked Grebes (Podiceps nigricollis).

It has been proposed that these patterns are the result of small-scale contact transmissions which are facilitated in spatially continuous water bodies. In contrast, wetlands, which are geographically separated, have to be bridged by bird movements [9]. This correlates with observations in Germany indicating that after the initial epidemics the overland spread of the virus bridging agricultural and forest areas was much slower.

There is increasing evidence indicating that influenza viruses can remain infectious in surface water for extended periods, especially at low temperature (between 4°C and 10°C) and low salinity [12], [13]. Influenza viruses may also accumulate in sediments of the littoral zone of shallow lakes [14]. This property may turn shorelines of lakes into infectious patches along flyways. For risk assessments regarding the spread of HPAIV H5N1 into new localities or poultry holdings after the introduction into wild bird population, a profound evaluation procedure of the wild bird monitoring at distinct geographical sites is necessary.

The principal aim of this study was to analyze the efficacy of the German avian influenza monitoring program in wild birds by a statistical evaluation model integrating the different aspects of wild bird monitoring data and incorporating external knowledge about bird species. We show that the presence of HPAIV H5N1 cannot be excluded for several temporal and spatial units despite intensive monitoring activities. The methodological approach is likely extendable and easily adaptable to other regions and pathogens.

## Methods

As a member state of the European Union, Germany is legally obligated to implement a surveillance program on avian influenza in wild birds (Article 4, Council Directive 2005/94/EC of 20 December 2005 on Community measures for the control of avian influenza and repealing Directive 92/40/EEC, following ‘Guidelines on the implementation of surveillance programs for avian influenza in wild birds to be carried out in the Member States’, Annex II, Commission Decision of 13 April 2007 on the implementation of surveillance programs for avian influenza in poultry and wild birds to be carried out in the Member States and amending Decision 2004/450/EC). All animal work was conducted according to these legal requirements and guidelines and all animals were handled in strict accordance with good animal practice as defined by the relevant national and/or local animal welfare bodies.

Apparently healthy birds were selected for testing by ornithologists, hunters or veterinarians following the national plan for avian influenza monitoring which allocates different minimum sample sizes to the German federal states and districts according to their area (active monitoring). Furthermore, deceased or sick birds were collected and tested for avian influenza (passive monitoring). Sampling is therefore not random and the sampled subpopulation is likely to be biased. Oropharyngeal and/or cloacal swabs were taken from live birds or carcasses and sent to each of the regional laboratories. Initial testing was performed by an Influenza-A generic rRT-PCR (M-PCR). M-PCR-positive samples were further analysed for H5-, H7- and N1-specific genome segments. In H5-positive samples, the HA cleavage-site that influences the pathogenicity of the isolate was determined by sequencing or by a specific rRT-PCR [15]. For the purpose of this study, only HPAIV H5N1-positive birds were considered as cases. The specificity of the monitoring system is assumed to be nearly 100% since positive test results are re-assessed by additional confirmatory and differentiating tests. The sensitivity of the system is determined by the sampling approach and the diagnostic sensitivity of the initial M-PCR.

For data collection, a national wild bird monitoring database was established. Central information storage was performed in a SQL database. An internet-server was installed for data-entry and descriptive inspection including maps and diagrams. A hierarchical bird identifier which is based on species level and comprises 430 different species allowed the precise identification of the birds in the database. If the species of a bird was not known, information at the family or genus level could be entered (40 families, 9 genera). For each bird that tested negative for avian influenza, the sampling site was recorded at the municipality level. For birds that tested positive for HPAIV H5N1 (‘cases’), the geographic coordinates of the sampling site were recorded. If the sampling date was missing, it was replaced by the data the sample arrived at the laboratory.

A statistical evaluation model was developed which is based on the determination of measurement sites as geographical features of interest for which an analysis of the monitoring is proposed. At these sites the prevalence (*p*) of HPAIV H5N1 cases in the sampled population of wild birds was smoothed in time and space. For each time (date) *t*, the prevalence *p_t_* is therefore the weighted sum of the corresponding diagnostic results:
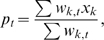
where *x_k_* is the diagnostic result of sample *k*, *d(x_k_)* is the date *x_k_* was sampled, and *w_k,t_* is the weight of *x_k_* at time *t*. 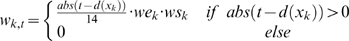


For this prevalence, the 95% upper confidence limit (UCL) was calculated, and for both, the estimated prevalence and the UCL, an analysis was performed for each measurement site. For time-dependent effects of different specimens, a smoothing window of 14 days pre- and post-sampling on the basis of a weighted moving average was implemented. The weighting considers specific coefficients for the spatial distance of the sample collection to the measurement site (*we_k_*) and the species selection (*ws*_k_). All calculations were done in R [16] (Text S1).

**Figure 2:**
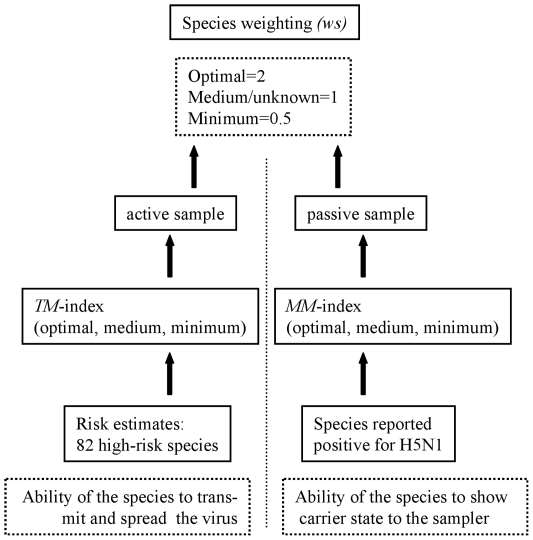
Weighting scheme for wild bird species.

For each bird, the lowest accepted spatial resolution of the sampling site was the municipality level which is on average 28.82 km^2^. The centroids of the municipalities were used to calculate ranges. For cases, the geographic coordinates of the sampling sites were used for distance determinations. Evaluation zones are defined for each measurement site as buffers which are limited by the time interval and the geographical range the monitoring database contained complete data records for. The distance (*B*) to the boundary of this buffer was used as an endpoint in the analysis. The distance between measurement and sampling site was determined using ArcGIS™ (ESRI^©^, Redlands, CA., USA). The analysis incorporated a linear weighting factor (*we*_k_) determined by the distance (*D*_k_) of the sampling to the measurement site. In this study the shorelines of the Baltic Sea, a freshwater lake near the town of Wusterhausen and the coordinates of an outbreak in a domestic duck holding were evaluated. Municipalities which are adjacent to the measurement site or whose centroid is within 2000 meters were assigned the value one (*we*_k_ = 1). All other municipalities were weighted by the following formula:
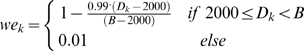



Confidence limits for prevalence estimations depend on the likelihood of virus detection in the tested individual. The probability of virus detection may depend on the susceptibility of different wild bird species and the health status of a bird at sampling. Therefore two species indices were introduced as weighting factors for the point and the interval estimator (Figure 2). Using a list of 82 susceptible species reported in 2007 [17], a transmission index (*TM*-index) was determined to evaluate the objectives of the active monitoring using the criteria listed in Table 1 as a rough estimate for the ability of a species to transmit and distribute HPAIV H5N1. A second index estimated morbidity and mortality (*MM*-index) due to HPAIV H5N1 infection in those species sampled by passive monitoring. The *MM*-index is considered a measure for the ability of a species to develop clinical signs in reaction to infection with HPAIV H5N1. The data sources for susceptible birds were the wild bird species known to be affected by HPAIV H5N1 in Germany and the species reported in the chart of affected species in the United States Geological Survey [18]. The indices were transformed for each sampling event in a weighting factor (*ws*_k_) used to classify their value for monitoring as optimal, medium or minimal. In the time series analysis different confidence limits were visualized to compare different scenarios. The individual weightings of the sampling event were compared to a minimal scenario, i.e. untargeted bird selection, and with an optimised scenario, where each sampling had an optimal value for monitoring.

**Table 1:** Criteria for the composition of two indices for wild bird species.

Indices	Definition
***TM*** ** = 1** “low”	(i) listed in Veen *et al* (all *Procellariiformes; Strigiformes; Prunellidae*; *Turdidae* (except the Trushes) and (ii) not considered as high risk species
***TM*** ** = 2** “medium”	(i) not listed by Veen *et al.* and (ii) no other information available
***TM*** ** = 3** “high”	82 high risk species
***MM*** ** = 1** “low”	No reference or information of cases of HPAIV H5N1 in this species
***MM*** ** = 2** “medium”	Species from the same family (resp. genera within *Anatidae* and *Accipitridae*) reported as cases infected with HPAIV H5N1
***MM*** ** = 3** “high”	(i) Species for which fatal H5N1 cases were reported from Germany or (ii) U.S.Geological Survey ( = 52 species)

*MM* = Index for Mortality and Morbidity; *TM* = Transmission-index.

Spatial autocorrelation was calculated in the R-package spdep using a contiguity neighbourhood weight for the municipalities in the study area.

Sample sizes were calculated from the statistical evaluation model using the formula of *Cannon and Roe* [19] for an arbitrary simulated measurement site:
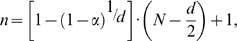
where *n* is the necessary sample size, *α* the error probability, *d* the number of positive animals and *N* the population size. The main assumptions for the statistical evaluation model are (i) a 100 km circular buffer including an average number of equally sized municipalities, (ii) a uniform distribution of sampling in space and time and (iii) an optimal species selection.

## Results

### Bird species coverage of the monitoring system

The dataset of the German wild bird monitoring comprised 16,554 entries for areas of complete coverage in 2006 and 25,545 entries for 2007. During this time, 217 different bird species were tested (Table 2). The proportion of birds with classification at the species level increased from 61.3% in 2006 to 81.7% in 2007. Active monitoring accounted for 52.9% on average. During the initial stage, the proportion of active monitoring increased from 28.4% in 2006 to 68.7% in 2007. Passive monitoring of deceased and sick wild birds accounted for 99.8% of all samples that tested positive for HPAIV H5N1 (Table 3). By active monitoring, only a single HPAIV H5N1 infected bird was detected [9]: a Mute Swan which was sampled 24 km from the Helme reservoir at Berga-Kelbra five days after the start of an outbreak in July 2007 that affected mostly Black-necked Grebes.

**Table 2:** Monitoring of different wild bird species and their status during sampling.

Year	Sample entries	Number of species	Species deter-mined	Type of monitoring						
				active			passive			Unknown
				alive	hunted	%	dead	sick	%	
**2006**	*16,554	165	**61.3%**	3,825	884	28.4	11,658	33	70.6	154
**2007**	25,545	190	**81.7%**	16,023	1,523	68.7	7,898	101	31.3	0
**total**	42,099	217	**73.7%**	19,848	2,407	52.9	19,556	134	46.7	154

* For data integrity reasons all analyses are restricted to the areas of complete coverage.

**Table 3:** Documented HPAIV H5N1 cases and bird species determination.

Year	Type of monitoring		Bird species determined	
	active	passive		
**2006**	0	224 (343*)	159 (160*)	71% (46.6%*)
**2007**	1**	326	320	98.2%
**total**	1**	550 (669*)	479 (480*)	87.1% (71.6%*)

* data for whole Germany in 2006.

** Mute Swan which was sampled in 24 kilometre distance five days after the outbreak at the Helme reservoir at Berga-Kelbra, 2007.

### Development of the monitoring in time and space

**Figure 3:**
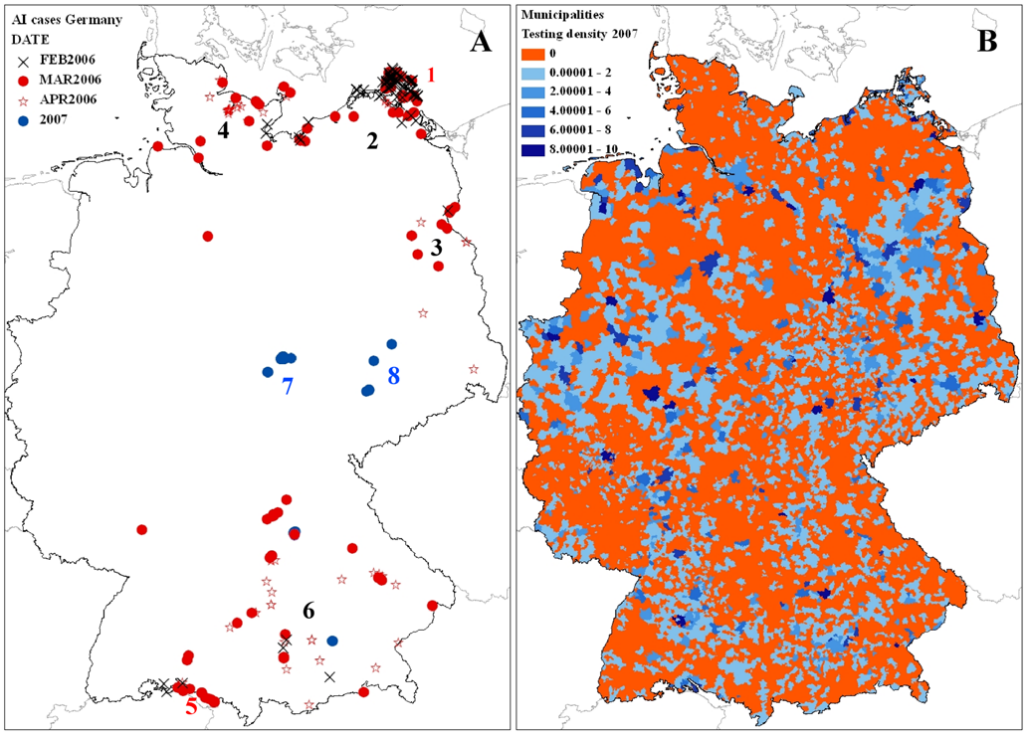
Results of the HPAIV H5N1 monitoring. Regions with cases of HPAIV H5N1 infection in wild birds in Germany (A). Spatial distribution of the wild bird monitoring in German municipalities 2007 (samples per square kilometer) (B). After the initial outbreak on the Isle of Ruegen (1), only three cases were recorded in inland municipalities at the coastline of Mecklenburg-Western Pomerania (2). Most of the cases in the neighboring states of Brandenburg (3) and Schleswig–Holstein (4) were reported several weeks afterwards in March and April 2006. HPAIV H5N1 cases at Lake Constance (5) were connected to further spread in smaller wetland areas in Bavaria (6). The massive outbreak in 2007 at the Helme reservoir at Berga-Kelbra (7) was related to four cases eastward in Saxony (8).

**Figure 4:**
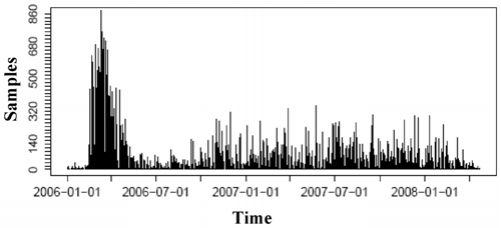
Distribution of wild bird samples. The daily number of wild bird samples tested for avian influenza is shown for the period 01 January 2006 until 01 March 2008.

Constant temporal and spatial sampling rates are required for mobile pathogens like HPAIV H5N1 to achieve a considerable degree of confidence in the results. Analysis of the temporal distribution of the sampling within the wild bird monitoring program showed, however, that the sampling rates decreased after an initial period of intensive testing upon detection of the index case and reached a more or less constant level (Figure 3). Short-term infrequent sampling produced unbalanced datasets with high autocorrelation in time, ranging between 0 samples on some days and up to 300 on others. The variation in space exhibited a similar pattern (Figure 4). The allocation of the monitoring activities to the different German federal states led to a nearly homogeneous distribution of the sample size over the entire country at the state level Measure of overall spatial autocorrelation: (Moran's I: 0.09; C-l: 0.08-0.1; expected value: −0.00008) [20]. Yet, irregular sampling at the district or municipality level produced data with considerable spatial dependencies. Sampling often concentrated around outbreak areas and on susceptible species. This prompted for instance high sample sizes at the Baltic Sea coast and at Lake Constance in reaction to outbreaks in these locations.

### Evaluation of monitoring activities by time series analysis

**Figure 5:**
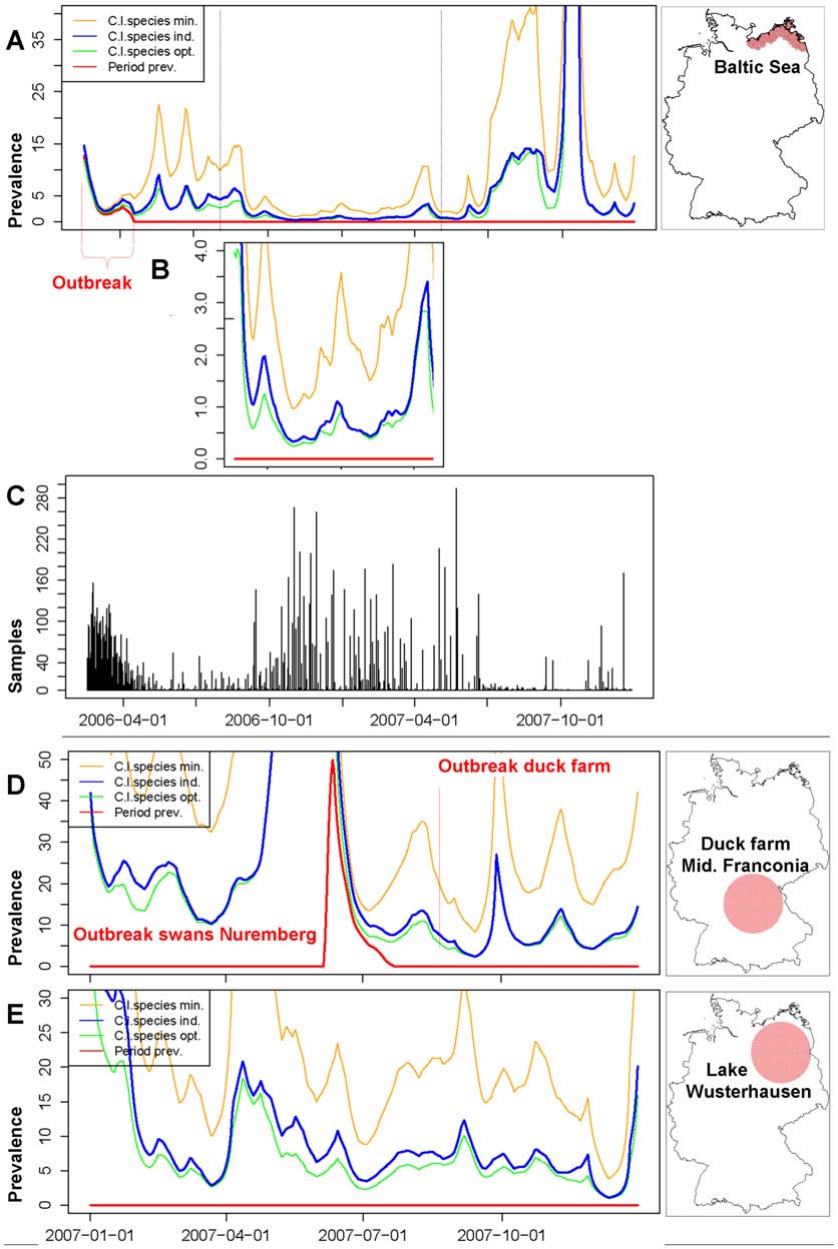
Time series analysis at different sites. Development of the period prevalence (red) and the upper confidence limit for comparing different species weighting (A) after the outbreak at the Baltic Sea coast. Detailed extract of the most intensive monitoring phase (B). Daily frequency of tests for the same time span in that region (C). Monitoring in a 118 km buffer around an outbreak of HPAI H5N1 in a domestic duck farm (D). Exemplary analysis in a region inside a 118 km buffer at a lake without cases of HPAIV H5N1 (Lake Wusterhausen) (E). The statistical evaluation model was weighted using individual weighting indices (blue) or by selection of samples with an optimal value of the weighting factor (green) and a minimal scenario where birds were completely untargeted selected for sampling (orange). The spatial buffer applied to the sea-shore is 34 km.

To assess the reliability of disease parameters, we estimated the prevalence of HPAIV H5N1 in wild birds at distinct measurement sites and calculated interval estimates of the virus prevalence which may have remained undetected. Immediately after the initial detection of HPAIV H5N1 at the German coast of the Baltic Sea and during the following spread, massive sampling of wild birds followed in this area (Figure 5, C). This led to high confidence in the prevalence estimates, but due to the bias caused by the sampling of certain bird species during the outbreak, more extensive virus circulation cannot be excluded (Figure 5, A). The last positive wild bird in this area was detected on April 4, 2006. Thereafter, a period of low sampling rates followed during spring and summer 2006. Only prevalences above a level of 3% to 8% can be excluded for this time span. Sampling increased again between September 2006 and May 2007 with the effect that the monitoring system was able to detect low prevalences between 15 October 2006 and 15 March 2007 with an UCL varying between 0.4% and 2.0% (Figure 5, B). Since no HPAIV H5N1 was detected during this period, it can be implied that the prevalence was below the respective threshold value in the area. Based on these estimates and their confidence intervals, it can be largely excluded that an outbreak occurred but remained undetected in this time span in the Baltic Sea region. After this period, the monitoring activity decreased again and was insufficient to exclude a prevalence of<5% in the Baltic Sea region between 01 July 2007 and 01 November 2007. At the same time, HPAIV H5N1 circulated in central and southern Germany as evident by a local epidemic among Black-necked Grebes at the Helme reservoir, as well as cases in two domestic duck holdings and in Mute Swans. As a second example, outbreaks of HPAIV H5N1 infections in a domestic duck holding detected on 25 August 2007 were studied [4]. Two months before the outbreak, infections with genetically closely related viruses were detected at a distance of 42 km mainly in Mute Swans in Nuremberg on 23 June 2007. Taking the farm as the focal point, the monitoring action in wild birds in a radius of 118 km was low before the first infection was detected in Nuremberg (Figure 5, D). Sampling increased after the case had been confirmed and led to a higher precision of the prevalence estimates. The UCL in the time interval between 23 June and 25 August 2007 varied between 7% and 13%, i.e. virus circulation in the wild bird population at relatively high levels could not be ruled out. In most parts of Germany, HPAIV H5N1 was not detected in 2006 and 2007. In these regions, the sampling procedures of the monitoring system could not be geographically linked to outbreaks. A freshwater lake near Wusterhausen, Brandenburg, was considered as an example for this situation (Figure 5, E). No outbreak was detected in this area and a high prevalence among wild birds can be excluded. For most time periods, a prevalence of<5% could not be ruled out, for some intervals the detection limit was as high as 10%.

### Species scoring

**Figure 6:**
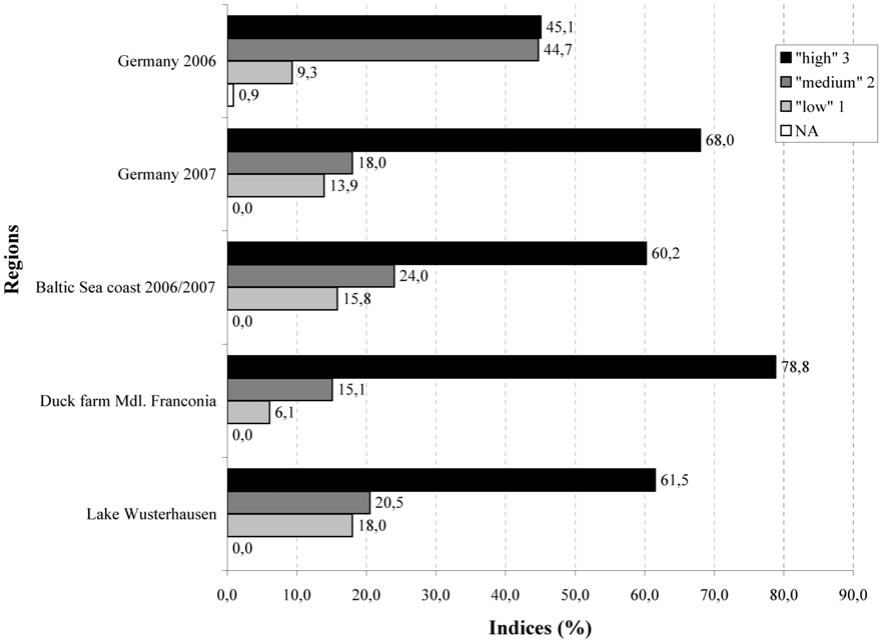
Distribution of indices at the time of sampling.

The species indices were retrospectively evaluated. During the monitoring in 2006, 45.1% of the samples could be regarded as ‘optimal’ (Figure 6) with respect to the criteria in the index for species selection (Table 1). The proportion increased to 68% in 2007. For the different measurement sites, the study area in Middle Franconia produced 78.8%, the Baltic Sea coast 60.2% and the zone around Wusterhausen 61.5% optimal index values. These index values were introduced into the statistical evaluation model as a weighting factor (Figure 5). The individual species indices were compared with an optimal and a minimum scenario for bird selection in the time-series analysis. For most intervals at the Baltic Sea coast, the individual scenarios could not be distinguished from the optimal scenario. A significant loss in detection power could not be observed. In other regions, considerable differences from the optimal scenario were evident, e.g. at Lake Wusterhausen during 2007. Therefore, improved bird species selection might have increased the confidence in the prevalence estimate in regions other than the Baltic Sea coast area.

## Discussion

Wild birds that carry and excrete HPAIV form a contact network and enable the virus to become a geographically highly mobile pathogen [21]. Due to their remoteness, varying populations and the migratory behavior of many wild bird species, these animals constitute a major challenge for designing disease monitoring and monitoring systems. A sound scientific analysis of data at the national and international level is required for risk assessments and scientific advice to risk managers [22], [23]. The German monitoring data show that only extensive transmission of the virus and a high prevalence can be excluded at some time points, for some species and some areas, where no case of HPAI H5N1 was found. In contrast, prevalences regularly reported for LPAIV for some wild bird species in other countries cannot be excluded with this system. Due to low sample sizes and partially untargeted sampling, the probability of detection of infected animals was low for most intervals and bird species.

In contrast to the passive monitoring, which only has the ability to detect virus in species that are infected sub-clinically, the sampling of apparently healthy birds (active monitoring) was unable to either detect or exclude transmission (Table 3). The only case detected by active monitoring suggested a radial virus distribution from the epicentre of an epidemic among Black-necked Grebes. Alternatively, the bird may have contracted the infection in the focus of the epidemic, moved and was sampled during the incubation period. Generally speaking, the active monitoring failed to prove or reject any hypotheses regarding specific species-related virus dissemination of HPAIV H5N1 in Germany.

The increase in precision in species selection is probably the result of improving knowledge of the sample collectors and an improved cooperation between diagnosticians and ornithologists. The indexing of species and the statistical evaluation model showed that the selection of specific target will improve the effectiveness of the monitoring program.

There are two main objectives for the determination of confidence limits for animal disease monitoring results. The first is related to quality assurance and describes whether a system can be used to verify or reject a hypothesis. The second objective is more related to empirical science and describes the proportion of the disease impact that remains undetected and may contribute to unnoticed spread of an infection. The evaluation procedure described in this paper for the monitoring of HPAIV H5N1 may prove useful for the detection of time intervals, geographical units and population subgroups where monitoring is insufficient to detect or exclude the presence of a pathogen at a low prevalence. Questions regarding the effect of single sampling events on precision and confidence limits remain open.

Since spatial dependencies can lead to a decrease in detection power of monitoring systems, especially in the short-term detection of an infectious pathogen introduced in large regions [24], precise population parameters are required for each region to obtain reliable estimates of the power of the monitoring system. As data on population sizes are lacking in wild animals, confidence limits specific for selected geographical measurement sites may provide an alternative for estimating the detection power for specific time intervals.

When the statistical evaluation model described here is applied to Germany as a whole, it can be calculated that 318 samples need to be tested daily to detect a prevalence of HPAIV H5N1 in wild birds exceeding 1% (Table 4). This corresponds to 116,070 samples per year which is more than 4-fold the sample size obtained in 2007. However, transmission of some LPAIV subtypes proved to occur far below this threshold [25], [26]. The same may also be true for HPAIV H5N1. Thus, installing a reliable monitoring system over a long period with limited resources still remains a major challenge.

**Table 4:** Sample sizes required for detection/exclusion of HPAIV H5N1 at selected design prevalence thresholds at the 95% confidence level.

Threshold	Daily sample size (municipality)	Daily sample size (Germany)
5%	0.006	69
1%	0.026	318
0.1%	0.100	1,244

The performance of a monitoring system is also reflected by the degree of confidence which is obtained by the data. This depends to a high degree on the assumptions on the pathogen to be monitored. HPAIV H5N1 proved to be highly-mobile and requires an improved targeted approach.

In the future, a scientifically sound and profound sampling of defined bird species at selected spots with reasonable sample sizes could lead to more reliable monitoring results with improved confidence. Furthermore ornithological as well as veterinary knowledge and infrastructure has to be integrated to develop a risk-based approach and to target particular bird species. A sensitive classification of species into risk categories and sampling individuals of these species at different time points along their flyways may aid in this respect. Moreover, time and location in relation to the migration routes of birds have to be taken into account. Participatory approaches involving local ornithologists can lead to an early detection of wild bird populations with increased density and unusual mortality and morbidity. Alternative monitoring approaches using sentinel birds, i.e. virus-negative birds that are closely monitored on a virological basis in areas of increased risk, can be related to local wild bird censuses and provide disease information for long time ranges [27]. Another possibility would be to focus on predator birds and scavengers which feed on a variety of diseased or dead birds leading to an increased probability to become exposed to HPAIV H5N1. As they proved to be susceptible to the virus, they could be a future target for improved monitoring in wild birds in Germany.

The evaluation model described in this study may help to analyze incoming monitoring data on avian influenza and to improve the targeting of monitoring programs. It should also be possible to apply it to other wildlife diseases.

## Supporting Information

Text S1 (0.05 MB DOC)
