# Supplementary material for: Chances and Limitations of Wild Bird Monitoring for the Avian Influenza Virus H5N1 — Detection of Pathogens Highly Mobile in Time and Space
Source: PLoS One. 2009 Aug 14;4(8):e6639. doi: 10.1371/journal.pone.0006639 (PMC2722081; doi:10.1371/journal.pone.0006639)
Supplement: Text S1 — (0.05 MB DOC) [file pone.0006639.s001.doc]

**Appendix:**

Code of the statistical evaluation model. The programming language is R (http://www.cran.r-project.org/).

#--------------------------------------------------------#

#

setwd("C:/RESULT")

#smoothing window

window<-14

startweighting<-2000

startvalue<-1

require(foreign)

### initialise graphics

grname<-paste('Prev_',window,sep='')

grname<-paste(grname,'Compare',sep='')

pdf(paste(grname,'.pdf',sep=''),paper='special',width=6,height=9)

par(mfrow=c(3,1),mar=c(5,5,3,1.5),cex.main=1.5,cex.lab=1.25,cex.axis=1.25)

### Species evaluation

speciesfile<-'C:/INDICES.dbf'

species<-read.dbf(speciesfile)

attach(species)

#--------------------------------------------------------#

# start function eval()

eval<-function(titel,number,basicfile,shorefile,yhauf)

{

##### data input

### shore communities

shore<-read.dbf(shorefile)

# rename designation

SHORE<-as.character(shore$AREA)

remove(shore)

### basic data

basis<-read.dbf(basicfile,as.is=T)

attach(basis)

ID<-as.character(ID)

AREA<-as.character(AREA)

date12<-DATE_

date12<-format(date12,"%Y%B")

# selection of distance vector

ee<-cbind(D_BALTIC,D_FRANCO,D_WUSTER,D_LAKECO)

distance<-ee[,number]

# designation off shore municipalities

rand<-rep(F,length(ID))

for (k in 1:length(SHORE)) rand[as.character(AREA)==SHORE[k]]<-T

cat('\n----------------\n',titel,'\n----------------\n')

cat('\nspecimen in Shore municipalities\n')

print(table(rand),quote=F)

cat('\ndistance shore municipalities\n')

print(summary(distance[rand]),quote=F)

cat('\nDistance to other municipalities\n')

print(summary(distance[!rand]),quote=F)

#distance weighting

value<-rep(startvalue,length(ID))

value[distance>= endweighting]<-endvalue

choice<-!rand & distance<endweighting

value[choice]<-startvalue+(endvalue-startvalue)/(endweighting-startweighting)*(distance[choice]-startweighting)

cat('\nWeighting for Distance\n')

print(summary(value),quote=F)

# summarisation species evaluation

scores<-rep(0,length(ID))

for (k in 1:length(SEQUEN))

{

scores[as.character(SPECIES)==as.character(KEYS[k]) & ACTIV==1]<-TM_INDEX[k]

scores[as.character(SPECIES)==as.character(KEYS[k]) & PASSIVE==1]<-MM_INDEX[k]

scores[as.character(SPECIES)==as.character(KEYS[k]) & is.na(ACTIV) & is.na(PASSIVE)]<-(TM_INDEX[k]+MM_INDEX[k])/2

}

cat('\nScores for Species individually\n')

print(table(scores),quote=F)

scores[scores==3]<-4

scores<-scores/2 # Mean (scores)

cat('\nnormed Scores for species\n')

print(summary(scores),quote=F)

###Weightings#

weighting<-scores*value # individual species weighting

weighting_opt<-2*value # optimal species weighting

weighting_min<-0.5*value # minimal species weighting

cat('\nWeighting factors complete\n')

print(summary(weighting),quote=F)

cat('Date\n')

date1<-DATE_

cat('first date\n')

print(min(date1),quote=F)

cat('last date\n')

print(max(date1),quote=F)

#--------------------------------------------------------

##### Prevalence and confidence limit

cat('specimen complete\n')

print(table(RESULT,exclude=NULL),quote=F)

quantdays<-as.integer(max(date1)-min(date1))+1

cat('quantity of days complete\n')

print(quantdays)

time<-1:quantdays

long<-length(time)

start<-min(date1)

time12<-rep(start,long)

for (k in 1:long) time12[k]<-start+k-1

quantspeci<-rep(NA,long)

for (k in 1:long) quantspeci[k]<-length(RESULT[date1==time12[k]])

cat('max. quantity of specimen each day\n')

print(max(quantspeci))

###optimal species weighting

prev_opt<-rep(NA,long)

conf_opt<-rep(NA,long)

for (k in 1:long)

{

choice<- abs(as.integer(time12[k]-date1))<=window

weight_opt<-weighting_opt*(window-abs(as.integer(time12[k]-date1)))/window

all_opt<-sum(weight_opt[choice])

if (all_opt>0)

{

posi_opt<-sum(weight_opt[choice]*RESULT[choice])

prev_opt[k]<-posi_opt/all_opt

conf_opt[k]<-prop.test(posi_opt,all_opt,alternative='less')$conf.int[2]

}

}

### minimal species weighting

prev_min<-rep(NA,long)

conf_min<-rep(NA,long)

for (k in window:long)

{

choice<- abs(as.integer(time12[k]-date1))<=window

weight_min<-weighting_min*(window-abs(as.integer(time12[k]-date1)))/window

all_min<-sum(weight_min[choice])

if (all_min>0)

{

posi_min<-sum(weight_min[choice]*RESULT[choice])

prev_min[k]<-posi_min/all_min

conf_min[k]<-prop.test(posi_min,all_min,alternative='less')$conf.int[2]

}

}

# individual species weighting

prev<-rep(NA,long)

conf<-rep(NA,long)

for (k in 1:long)

{

choice<- abs(as.integer(time12[k]-date1))<=window

weight<-weighting*(window-abs(as.integer(time12[k]-date1)))/window

all<-sum(weight[choice])

if (all>0)

{

posi<-sum(weight[choice]*RESULT[choice])

prev[k]<-posi/all

conf[k]<-prop.test(posi,all,alternative='less')$conf.int[2]

}

}

##### Graphics

plot(prev~prev,type='n',axes=F,xlim=c(0,100),ylim=c(0,100),xlab='',ylab='',main='')

text(50,80,titel,cex=2.5)

text(50,40,paste('Time window',window,'Days'),cex=1.75)

text(50,20,paste('Comparison of species weight (Scenarios)\nDistancesweighting individual'),cex=1.75)

#-----#

plot(100*prev~time,type='l',axes=F,ylim=c(0,axisvalue1),col='red',lwd=2,

xlab='Time',ylab='Prevalence',main='period prevalence and upper confidence limit (UCL)')

lines(100*conf_min~time,col='orange',lty=1,lwd=1)

lines(100*conf_opt~time,col='green',lty=1,lwd=1)

lines(100*conf~time,col='blue',lwd=2)

box()

quarter<-substr(time12,9,10)=='01' & (substr(time12,6,7)=='01' | substr(time12,6,7)=='04' | substr(time12,6,7)=='07' | substr(time12,6,7)=='10')

axis(1,time[quarter],time12[quarter])

axis(2,seq(0,axisvalue1,axisvalue2))

legend('topleft',legend=c( 'C.l. species min.', 'C.l. species ind.', 'C.l. species opt.','Period Prev.'),lty=1,col=c('orange','blue','green','red'))

#-----#

plot(quantspeci~time,type='h',axes=F,ylim=c(0,max(yhauf)),xlab='Time',ylab='Specimen quantitity',main='daily specimen frequency')

box()

axis(1,time[quarter],time12[quarter])

axis(2,yhauf)

#--------------------------------------------------------

detach(basis)

#dev.off()

} # end function eval()

##########################################################

titel<-'Baltic Sea'

tag<-'Baltic'

endweighting<-34000

endvalue<-0.01

number<-1

axisvalue1<-40

axisvalue2<-5

basicfile<-'C:/BALTIC_CASES.DBF'

shorefile<-'C:/BALTIC_SHORE.DBF'

eval(titel,number,basicfile,shorefile,seq(0,300,20))

#--------------------------------------------------------#

titel<-'Outbreak duck farm'

tag<-'Duckfarm'

endweighting<-118000

endvalue<-0.01

number<-2

axisvalue1<-50

axisvalue2<-5

basicfile<-'C:/FRANCONIA_CASES.DBF'

shorefile<-'C:/ FRANCONIA_SHORE.DBF'

eval(titel,number,basicfile,shorefile,seq(0,200,10))

#--------------------------------------------------------#

titel<-'Wusterhausen'

tag<-'Wuster'

endweighting<-118000

endvalue<-0.01

number<-3

axisvalue1<-30

axisvalue2<-5

basicfile<-'C:/WUSTERHAUSEN_CASES.DBF'

shorefile<-'C:/WUSTERHAUSEN_SHORE.DBF'

eval(titel,number,basicfile,shorefile,seq(0,220,10))

#--------------------------------------------------------#

titel<-'Lake Constance'

tag<-'Const'

endweighting<-34000

endvalue<-0.01

number<-4

axisvalue1<-80

axisvalue2<-10

basicfile<-'C:/LAKECONSTANCE_CASES.DBF'

shorefile<-'C:/ LAKECONSTANCE_SHORE.DBF'

eval(titel,number,basicfile,shorefile,seq(0,125,20))

#--------------------------------------------------------#

dev.off()
